# Constructing a database of alien plants in the Himalaya to test patterns structuring diversity

**DOI:** 10.1002/ece3.10884

**Published:** 2024-02-09

**Authors:** Suresh K. Rana, Bhawana Dangwal, Gopal S. Rawat, Trevor D. Price

**Affiliations:** ^1^ G.B Pant National Institute of Himalayan Environment Almora Uttarakhand India; ^2^ Wildlife Institute of India Dehradun Uttarakhand India; ^3^ Zoology 309A, Department of Ecology and Evolution University of Chicago Chicago Illinois USA

**Keywords:** alien species, climatic niche, directional filtering, elevational distribution, Himalaya, seed plants, species richness

## Abstract

Differences in the number of alien plant species in different locations may reflect climatic and other controls that similarly affect native species and/or propagule pressure accompanied with delayed spread from the point of introduction. We set out to examine these alternatives for Himalayan plants, in a phylogenetic framework. We build a database of alien plant distributions for the Himalaya. Focusing on the well‐documented regions of Jammu & Kashmir (west) and Bhutan (east) we compare alien and native species for (1) richness patterns, (2) degree of phylogenetic clustering, (3) the extent to which species‐poor regions are subsets of species‐rich regions and (4) continental and climatic affinities/source. We document 1470 alien species (at least 600 naturalised), which comprise ~14% of the vascular plants known from the Himalaya. Alien plant species with tropical affinities decline in richness with elevation and species at high elevations form a subset of those at lower elevations, supporting location of introduction as an important driver of alien plant richness patterns. Separately, elevations which are especially rich in native plant species are also rich in alien plant species, suggesting an important role for climate (high productivity) in determining both native and alien richness. We find no support for the proposition that variance in human disturbance or numbers of native species correlate with alien distributions. Results imply an ongoing expansion of alien species from low elevation sources, some of which are highly invasive.

## Abstract 2

Several reasons have been proposed for why alien species richness patterns vary across the globe, but they are rarely compared in a comprehensive test. Here we build a complete database of Himalayan alien plant species and show that both location of introduction and climate per se are likely important in explaining elevational and geographical patterns of species diversity.

## 1 INTRODUCTION

Alien species have been introduced to new areas for centuries. The process has increased exponentially in the past 50 years, with no signs of slowing down (Hulme et al., 2009; Sax et al., 2007; Seebens et al., 2017; Van Kleunen et al., 2015). However, the number of alien plant species varies across the globe, with some locations being relatively devoid of alien species, while others are heavily dominated by them (Guo et al., 2021; Pyšek et al., 2017). Here we evaluate the underlying causes of the distribution of alien species richness across the Himalaya. In order to do this we compare native and alien species richness, given different processes affect each. For example, differential rates of speciation can affect the richness patterns of native species, but not that of aliens (Sax, 2001).

The predominant pattern for native plant species richness is that warm and wet locations harbour more species than cold and dry ones (Francis & Currie, 2003; Kreft & Jetz, 2007). The pattern correlates with higher plant productivity (Currie et al., 2004; Hutchinson, 1959), aseasonality (Gao & Liu, 2018) and/or historical patterns of turnover (speciation and extinction) (Igea & Tanentzap, 2020; Rana et al., 2022), all of which are correlated with each other across space. In contrast, at a large spatial grain size, alien species richness broadly correlates with native richness, but unlike native richness, which is highest in the tropics, alien richness is thought to reach a maximum at mid‐latitudes (Guo et al., 2021; Sax, 2001). The alien pattern plausibly reflects a lack of knowledge of tropical introductions (Chong et al., 2021; Nuñez et al., 2022), but postulated biological processes include invasion resistance by species‐rich tropical communities, differences in disturbance and differences in number of introductions (Chong et al., 2021; Guo et al., 2021). Similarly, across elevational gradients alien richness broadly correlates with native richness because both decline towards high altitudes (Alexander et al., 2011) but sometimes alien richness shows a steeper decline than natives (e.g., Mallen‐Cooper & Pickering, 2008). Punctuating the decline with elevation, both aliens and natives may also show intermediate peaks in richness. The positions of the alien and native peak may roughly correspond (e.g., Denslow et al., 2010; Khuroo et al., 2011) or be displaced (Manish, 2021). The drivers of similarities and difference between natives and aliens along elevational gradients have yet to be assessed.

If native and alien patterns are concordant, and both strongly correlated with climate, a parsimonious inference is that climatic conditions in the introduced region are an important determinant of alien distributions (Sax, 2001). A corollary of this hypothesis is that if (1) climate per se is important and (2) native and alien species with similar climatic niches are closely related, we expect to observe increased phylogenetic clustering (the extent to which species are drawn from a few clades) when alien and native species are considered together, rather than separately (Qian & Sandel, 2022).

Deviations of alien patterns from native patterns suggest additional forces beyond climate are at play, which may apply to native species or to alien species. For example, native diversity in the Himalaya is affected by differential patterns of extinction and speciation in different climatic zones, as well as limitations on dispersal (Muellner‐Riehl, 2019; Rana et al., 2022), whereas as noted above alien patterns cannot be affected by speciation processes (Sax, 2001). A steep decline in alien richness across space may reflect location of introduction and the subsequent time it takes to extend range, whereas time available has been much longer to native species. When applied to mountains, a role for location of introduction coupled with a time delay is known as the directional filtering hypothesis, specifically invoked to explain a decline in alien richness from low to high elevations (the location of introduction is considered to be primarily at low elevations, Alexander et al., 2011; Marini et al., 2013). The directional filtering hypothesis predicts a pattern of nestedness, whereby species at higher elevations represent a subset of those at lower elevations (Alexander et al., 2011).

If directional filtering operates, both biotic and abiotic factors may limit range expansions. The contrasting roles of biotic and abiotic factors have been invoked to explain patterns of phylogenetic distance between alien and native species across the North American flora, as observed when moving from benign to harsh climates, and from small to large scales. The logic is abiotic factors lead to small phylogenetic distances (relatives are similarly adapted) and biotic ones to larger phylogenetic distances (close relatives exclude each other (Park et al., 2020)). Finally, disturbance, by removing competitors can facilitate alien establishment and is associated with introductions: e.g., exotic crops associated with agriculture.

In this paper, we study the distribution of Himalayan plants to evaluate hypothesised factors that account for variation in alien species richness. First, we develop a comprehensive database of Himalayan alien species, describe their source affinities and characterise their elevational ranges and geographical distributions within the Himalaya. Second, we use this compilation to assess underlying correlates of alien richness patterns. We evaluate causes of patterns of alien species richness across the Himalaya, as far as is possible with the resolution available, which is at broad spatial grain size (200 m elevational bands across large regions). Because this is a large grain size, we focus on elevational distributions in two relatively small geographically separated regions, one in the west (Jammu & Kashmir) and the one in the east (Bhutan, north Bengal and Sikkim), which are relatively well known and provide a summary of the species diversity gradients across space. Specifically, our goals are to:

1. examine a role for climate in affecting the distribution of alien plant species in the Himalaya by searching for correlates with climatic variables, and secondarily, by testing if alien and native species when considered together show relatively high phylogenetic clustering.
2. evaluate the evidence for directional filtering by asking if high elevation assemblages of alien species are nested within lower elevation assemblages, and if alien species show a pattern of nesting that is stronger than observed for native species. We also consider a possible role for a varying intensity of competition along the gradient as a driver of the patterns by testing if (a) the proportion of alien species declines with the number of native species, (b) increased phylogenetic clustering of alien species correlates with elevation, expected if abiotic conditions become more important in harsher climates (Park et al., 2020) and (c) highly disturbed areas contain more alien species.

## 2 MATERIALS AND METHODS

### 2.1 Data curation

We curated the information on distribution and elevational ranges of all alien species reported from the Himalaya in 31 regional floras and 93 research articles (Appendix S2). To compare the alien distribution patterns with natives, we used our previously published dataset on distributions of native plant species across the Himalaya (Rana et al., 2022; Rana, Price, et al., 2019). To harmonise taxonomy across sources, we assessed all species names against the updated list of botanical names available from www.worldfloraonline.org (World Flora Online) and resolved every taxon to the species level. Any species whose native distribution extends into the Himalayan region is considered as a native, whereas a species whose native range is elsewhere is considered as alien. Native and alien distributional ranges of all species were validated from various online sources including the Invasive Species Compendium (www.cabi.org/isc), the Global Invasive Species database (http://www.iucngisd.org/gisd/) and the USDA‐GRIN (https://npgsweb.ars‐grin.gov/). Complete dataset of alien species along with the hyperlinks to the sources are provided for each species in Appendix S3. Species were classified as cultivated (species that are cultivated by humans for food or ornaments) or naturalised (a species that does not require human assistance to reproduce and maintain itself in its natural environment). Note that many cultivated species may also be naturalised, but we prioritise cultivated over naturalised in this compilation. We also listed each species as invasive (species considered to have harmful impacts) as classified by the Global Invasive Species databases (hence not necessarily invasive in the Himalaya).

To assess source regions we recorded the native range of alien species to the continents (Africa, Australia, Europe, North America and South America; following Brummitt, 2001). Tropical Asia and Temperate Asia were not separated because the Himalaya acts as a boundary between these two regions and species distributions have been assigned too crudely to classify them unambiguously. Plant species were also classified into three biogeographic categories, i.e., tropical, temperate and cosmopolitan, based on the centre of species diversity within each genus, as categorised by Wu (1991). These data are unavailable for 525 species (<5%, 378 native species, 147 alien species) which we excluded from relevant analyses.

### 2.2 Species distributions

**FIGURE 1:**
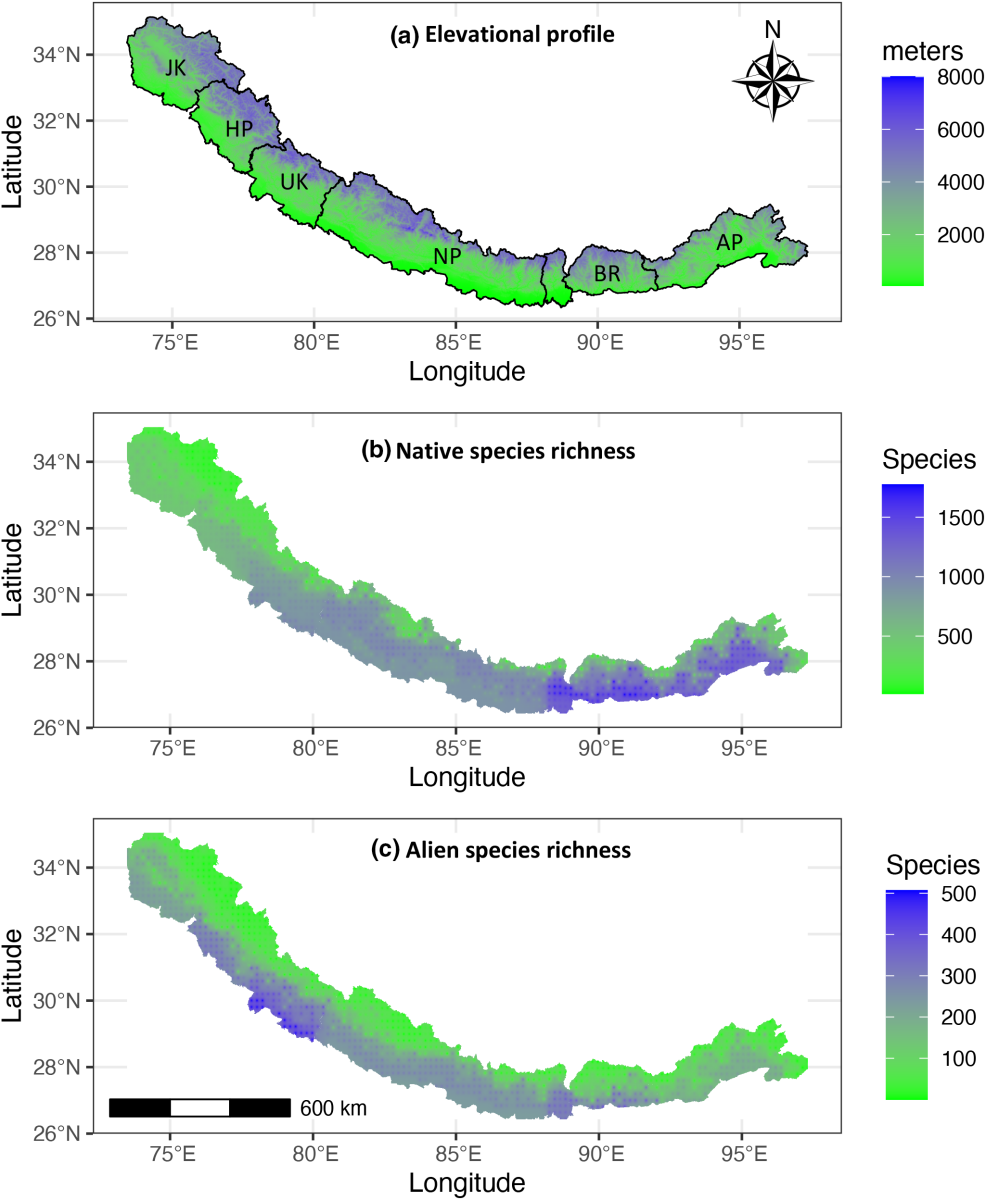
Maps of the Himalaya showing (a) elevations, with boundaries of the six regions we studied (JK: Jammu & Kashmir, HP: Himachal Pradesh, UK: Uttarakhand, NP: Nepal, BR: Bhutan region, AP: Arunachal Pradesh), (b) richness of native plant species, (c) richness of alien plant species.

The distribution of every species was categorised into six distinct regions from east to west, i.e., Arunachal Pradesh, Bhutan plus Sikkim and adjoining hill districts of West Bengal (henceforth the Bhutan region), Nepal, Uttarakhand, Himachal Pradesh and Jammu & Kashmir (Figure 1). To compare the elevational patterns between the east and west Himalaya, as noted above we particularly focus on two well‐known regions: Jammu & Kashmir (west) and the Bhutan region (east). To help interpret elevational patterns, we also assessed the distribution of different life forms (i.e., trees, shrubs, herbs, woody climbers, herbaceous climbers and epiphytes).

We considered only angiosperms (8765 native species and 1470 alien species). Elevational ranges of 1141 species (845 native species and 296 alien species) are unavailable and were excluded from the distributional analyses. Along elevational gradients, we calculated species richness as the number of species with overlapping ranges in every 200 m elevational band up to 5000 m in east and west Himalaya (Rana, Price, et al., 2019). To interpolate species richness patterns across the Himalayan landscape, we divided a Digital Elevation Model (https://srtm.csi.cgiar.org) into grids of size 0.2 degree (1140 grids). Within each of the six regions, grids at 0.2° resolution are assigned the presence of a species if it is recorded in that region, and its elevational range intersects the mean elevation of the cell. Species richness across the Himalaya was smoothed from these data by using the inverse distance weight interpolation method in the ‘gstat’ R package (Gräler et al., 2016).

### 2.3 Climatic data

We confined our analysis to annual precipitation (BIO12) and minimum temperature of the coldest month (BIO6), because of the apparent importance of freezing in setting the altitudinal distribution of native plant in the Himalaya (Rana et al., 2022). We obtained these climatic raster layers from the WorldClim (www.worldclim.org) database (Fick & Hijmans, 2017), using the ‘raster’ (Hijmans, 2017) and ‘sp’ (Bivand et al., 2013; Pebesma & Bivand, 2005) packages in R. Coordinates of the grids of size 0.2 × 0.2 degrees were used to extract annual precipitation and minimum temperature of every grid for further analyses. For natives plant species we have previously shown that, WorldClim and the alternative Chelsa dataset (Karger et al., 2017) give similar results across the Himalaya.

### 2.4 Phylogenetic analysis

We evaluate patterns of phylogenetic clustering to ask if (1) clustering is higher when native and alien species are considered together than when native species are considered alone, as predicted if related species have similar climatic niches and climate is an important control on establishment (Qian & Sandel, 2022) and (2) document patterns of phylogenetic clustering of native and alien species along the elevational gradient. To do this, all species were linked to a phylogeny implemented in the ‘V.PhyloMaker’ R package (Jin & Qian, 2019). The phylogeny was derived from two published trees (Smith & Brown, 2018; Zanne et al., 2014) and includes 74,533 species and all families of extant vascular plants (Jin & Qian, 2019). A total of 4579 Himalayan species are present in the backbone. To graft unresolved species (55%) and genera (26%), we used the ‘scenario 3’ approach implemented in ‘V.PhyloMaker’ (Jin & Qian, 2019). This method attaches a species of an existing genus to the basal node of the genus (if only one sequenced species is available for the genus, the basal node is generally placed half the distance from the family basal node to the tip), and any new genera to the basal node of the family branch.

Using the phylogeny and species presence/absence matrix for every 200 m elevational band, we calculated the mean pairwise distance (MPD) and mean nearest taxon distance (MNTD) (Webb et al., 2002) separately for the Bhutan region in the east and Jammu & Kashmir in the west, using the ‘PhyloMeasures’ R package (Tsirogiannis & Sandel, 2016). The phylogenies used for calculation of these metrics were restricted to alien species, native species or all plants together within these two focal regions. We computed the standardised versions of MPD and MNTD, i.e. the Net Relatedness Index (NRI) and Nearest Taxon Index (NTI), respectively, because these should be largely uncorrelated with species richness in null model phylogenies (Tsirogiannis et al., 2012). A positive value for NRI or NTI implies clustering (i.e., relatively few lineages contribute to the species present in a particular elevational belt), whereas a negative value implies over‐dispersion (i.e., many lineages distributed throughout the phylogeny); significance at *p* = .05 is equivalent to NRI (or NTI) > +1.96 (or < −1.96) (Mazel et al., 2016). We also computed species to genus ratios in 200 m bands (high ratios would be equivalent to clustering). To remove any biases due to differences in the number of genera between native species and alien species, we rarefied the native genera in each band to the same number as the alien genera (100 random samples) and show the mean value for both the rarefied and complete data.

### 2.5 Species nestedness

To test for directional filtering, we computed nestedness of alien and native plant species assemblages (Almeida‐Neto et al., 2008; Ulrich et al., 2009). First, we calculated the NODF*c* statistic (‘Nestedness metric based on Overlap and Decreasing Fill, standardised for Connectance’, Song et al., 2017), using the R package ‘maxnodf’ (Hoeppke & Simmons, 2021) which is designed to control for network size. We calculated NODF*c* for species occurrences in elevational bands of 200 m as one traverses from low to high elevations in Jammu & Kashmir (*N* = 23 bands) and the Bhutan region (*N* = 26 bands), as well as from west to east across the Himalaya at low elevations (below 1000 m). We also computed a phylogenetic version of NODF*c* using treeNODF (Melo et al., 2014) in the R package ‘CommEcol’ (Melo, 2021) which accounts for branch lengths. We test the hypothesis that the composition of species‐poor, high‐elevation sites are nested within the composition of species‐rich, low‐elevation sites, and that this pattern is stronger in aliens.

We developed a simple statistical test to compare nestedness between alien species and native species, which involved for each elevational band drawing 100 random samples from the native pool in the region, and 100 samples from the alien pool in the region, with the sample size set to the number of species present in a given elevational band. The sampling process revealed that with such a large number of species, the probability that native and alien species are drawn from a common pool is extremely low (difference in NODFc values greater than 0.05 are highly significant). Confidence limits are not reported given the large differences in observed NODFc values.

### 2.6 Disturbance measures

Both large numbers of native species and low levels of disturbance have been invoked as factors limiting establishment of aliens. Both operate at small grain sizes, whereas our analysis is of necessity at a large grain size, so we use our results only as a guide to possible limits to establishment. Following Beaury et al. (2020), we regressed the proportion of alien species in an elevational band on the number of natives (a negative slope may indicate inhibition of alien establishment by natives). We discuss the assumptions involved in applying this across large grain sizes in the supplement (Methods S1), where we show a linear model is more appropriate than the general linear model recommended by Beaury et al. (2020, see Figure S1). As a measure of disturbance, we use Venter et al.'s (2016) estimate of the human footprint for 2009, which is based on quantity of pastureland, agricultural land, night lights and built environments. Understory forest management, forest conversion to plantations and extensive grazing in native habitats are not recorded. We note that disturbance has been ongoing for thousands of years in the Himalaya and every elevational belt has been disturbed to some degree (Figure S2). Nevertheless, disturbance is greater in the west than the east Himalaya (Figure S2), as is apparent to visitors to each area.

### 2.7 Multivariate analyses

Having conducted the tests of hypotheses separately, we evaluated roles for some of the variables after statistically holding the others fixed, as many, including both climatic variables and disturbance correlate with elevation (Table S1). We asked if disturbance remained an important predictor of alien success after including number of native species and climate variables in a linear model assuming normal errors, and if climate or elevation per se better predict alien richness. In these analyses, we also included tests for interactions. We confirmed residuals were approximately normally distributed and homoscedastic through visual plots. Multi‐collinearity is not a problem in these tests (Table S1). We conducted various analyses to evaluate the robustness of the results to spatial area and connectivity, including using 1140 grids of 0.2° resolution across the entire dataset, and random sampling down to just 10–20 grids of 0.2°. All analyses gave similar results, and here we present regressions of numbers of alien species for entire 200 m elevational belts below 4000 m in the Bhutan region and Jammu & Kashmir. We excluded the 4000 m to 5000 m belt because very few alien species are present at these elevations.

## 3 RESULTS

### 3.1 Species richness patterns

In total, we documented 1470 alien angiosperm species, or approximately 14% of the known Himalayan flora. Of these, 614 are considered completely naturalised, which is twice the number in a global database (van Kleunen et al., 2019), but similar to that which comes from a recent study based on checklists from the relevant states (Wani et al., 2023). The van Kleunen et al. (2019) database reports 606 naturalised alien species in the Himalaya, but in our appraisal only ~50% of these species are alien, and the remainder are native.

Among associations with different continents, North America provides the most alien species (402), followed by South America (388), Europe (368), Africa (310) and Australia (106). Note that the total is greater than 1470 because some alien species are native to multiple continents. The pattern for alien species is almost the inverse of that for native species (native species: Africa [519 species] followed by Europe [341], Australia [347], North America [132] and South America [88]).

**FIGURE 2:**
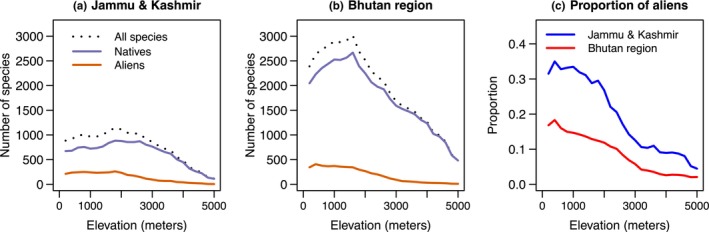
Species richness pattern of all species, native and alien species in 200 m elevational belts in Bhutan region and Jammu & Kashmir, and, *left*, the proportion of alien species in each belt.

Species richness for both aliens and natives decreases from the east to the west Himalaya (Figures 1b,c and 2). Jammu & Kashmir contains 70% fewer native species than the Bhutan region (1649 vs. 5452), respectively (Rana, Price, et al., 2019) but only 16% fewer alien species (556 vs. 634). Along elevational gradients in belts of 200 m, native and alien species shows a strong positive correlation both in the west and the east Himalaya i.e., *r* = .79 (Jammu & Kashmir) and *r* = .92 (Bhutan region, see Figure 2). Alien species richness remains approximately constant up to 2000 m, and then declines with elevation (Figure 2), a feature common to both naturalised and cultivated alien species (Figure S3), as well as different life forms (trees, shrubs, herbs; Figure S4). The elevational decline associated with the 762 cultivated alien species, many of which are naturalised as well, may reflect the large number of species cultivated across the plains of India, which are also present in the Himalayan foothills. However, the 614 completely naturalised species show a similar decline (Figure S3).

**FIGURE 3:**
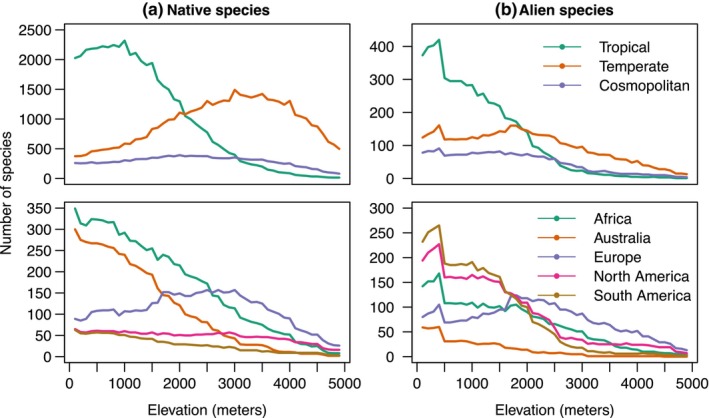
Elevational richness patterns of angiosperm plant species in the Himalaya assessed every 200 m, as the number of species whose range overlaps that elevation, split by biogeographical affinities. Top panels refer to species categorised by Wu (1991) as belonging to tropical or temperate genera, and lower panels to species shared between the Himalaya and other regions. Left: native species, right: alien species.

Alien species richness declines with elevation for taxa with tropical affinities, whereas those with European/temperate affinities show a peak at 2000–3000 m (Figure 3). Native species with tropical affinities show a mild peak at 1000 m, and those with temperate affinities a more pronounced peak above 3000 m. Thus, the peak is about 1000 m higher for native species than for alien species, both for species with tropical and temperate affinities.

### 3.2 Climatic control

**FIGURE 4:**
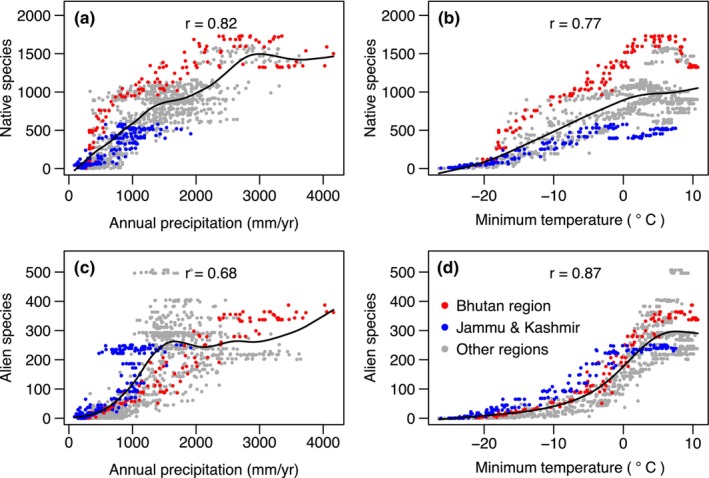
Scatter plot of species richness verses climatic variables (annual precipitation and mean minimum temperature of the coldest month) for 1140 grids of 0.2° resolution across the Himalaya, based on species richness maps in Figure 1. Alien species lower row, native species upper row. Lines are nonparametric spline fits.

Correlations between alien and native richness are at least in part a result of both being correlated with climate (Figure 4). Climatic controls are particularly apparent from the distribution of species with tropical and temperate affinities. In the subtropical Bhutan region, 48% of the native species and 52% of the naturalised alien species have tropical affinities, whereas in the drier and more temperate Jammu & Kashmir the corresponding figures are 31% and 32%. The similarity between the native and alien species is striking and suggests that present climate is an important determinant of alien species richness.

**FIGURE 5:**
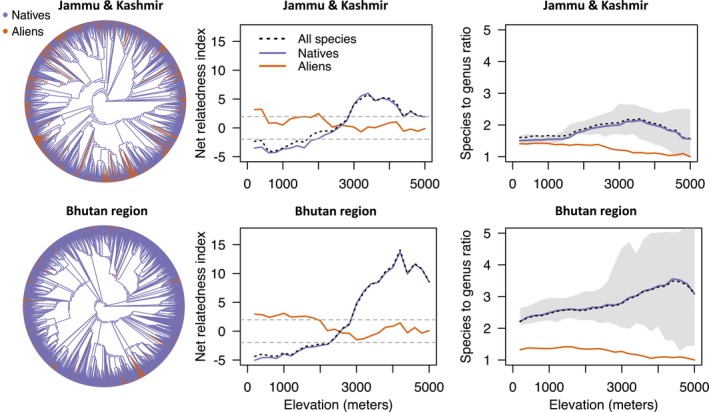
Left: Phylogenies of 5452 native and 634 alien species in Bhutan and 1649 native and 556 alien species in Jammu & Kashmir showing tips of the two groups in different colours. Centre: Net Relatedness Index of all species, native species, and alien species separately along 200 m elevational belts in Jammu & Kashmir (alien: native correlation, *r* = −.64) and Bhutan region (alien: native correlation, *r* = −.65). Dashed lines are ±1.96, i.e., values outside this limit are putatively significant at *p* = .05 (ignoring multiple testing issues). Native and alien indices are based on subsets of the phylogenies on the right (for similar Nearest Taxon Indices, see Figure S5). Right: Species to genus ratios for native and alien species along 200 m elevational belts in Jammu & Kashmir and Bhutan region. Black dotted line is the mean based on subsampling the native species pool down to that of the alien species numbers in the belt (shading indicates 95% confidence limits). Figure S6 shows species:genus ratios subset according to tropical and temperate affinities.

A possible additional test of a role for climate comes from assessment of the net relatedness index, which is predicted to increase in a combined dataset as compared to only native species, under the assumption that those alien species with similar climatic niches to native species are also closely related to them (e.g., Qian & Sandel, 2022). In fact, the net relatedness index of native species across elevational gradients barely differs from that of alien species plus native species combined (Figure 5).

### 3.3 Directional filtering

**FIGURE 6:**
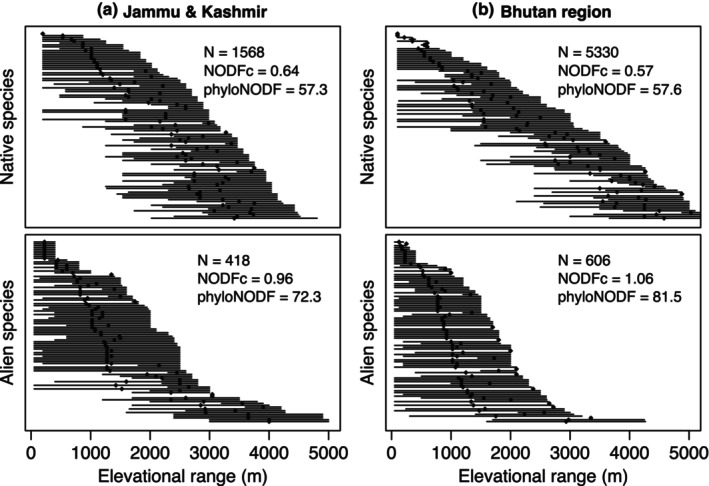
Elevational ranges of 100 randomly drawn species (lines) and mean elevation of occurrence (points). The nestedness statistic is calculated for all species (*N*) in each region (as in Table S1). Statistics include both phylogenetic and non‐phylogenetic measures.

We compared nestedness in species composition across elevations. In all comparisons, alien species are much more nested than native species, with differences ranging from one‐third to twice as much (Figure 6; Table S2). Alien plant species with tropical affinities have values of nestedness 20% higher than species with temperate affinities (Table S2), which is expected if sources of introduction for those with tropical affinities are from the bottom of the mountain, but those with temperate affinities are often at higher elevations.

While it is not straightforward to include the nestedness metric in the regression models, we find that in a model with climate and elevation together, the significance of climate is greatly reduced (Tables S3 and S4), which we interpret to be because elevation integrates the combined effects of both directional filtering and climate on alien richness.

### 3.4 Limits to establishment

The proportion of alien species weakly increases with disturbance (Table S1) but disturbance disappears as a factor when elevation is included in multiple regression models (Table S3). The proportion of alien species increases with number of native species (Figure S7), providing no evidence that number of native species inhibits establishment of aliens.

## 4 DISCUSSION

### 4.1 Species richness patterns

Here we have presented a comprehensive database of alien plant species in the Himalaya, extending a recently published database which documents 771 alien species, and shows that they have wider geographical ranges, on average, than natives (Wani et al., 2022, 2023). We document 1471 alien species (14% of the described flora), and add estimates of elevational ranges. Of the alien species, one‐third are entirely naturalised, and half of the naturalised species are considered invasive at least somewhere in the world, according to the Global Invasive Species database (235 invasive species). Our results suggest some previous generalities might need re‐evaluation, at least for Asia, such as the presence of more alien species in temperate relative to tropical climates (e.g., Richardson & Pyšek, 2012; Van Kleunen et al., 2015).

A striking finding is that the highest number of Himalayan alien species come from the Americas, whereas the highest number of native species are shared with Africa, Europe and Australia (as reported for a subset of alien species in India (Khuroo et al., 2012; Pandit, 2017, pp. 243–245) and China (Feng et al., 2011; Liu et al., 2006)). It is possible that the record of relatively few alien species from Africa and Australia represents misclassification of alien species as native species, given the long periods of time over which human exchange has been ongoing (e.g., Boivin et al., 2016; Demske et al., 2016) but the disparity in numbers seems too great to account for the alien/native species difference in sources and affinities. We presume that the preponderance of alien species from the Americas is a consequence of introduction biases. That presumption is supported by the global analysis of Turbelin et al. (2017), who noted that about two‐thirds of >880 invasive plant species had been deliberately introduced for agriculture or horticulture. Other species have been carried along as weeds (Boivin et al., 2016). Khuroo et al. (2012) briefly review historical trade since the 16th century that might have led to the large number of introductions from the Americas. Present global databases classify many Himalayan natives as aliens, and this plus the different affinities of natives and aliens suggest that inferred historical drivers of alien presence need to be re‐appraised (cf. Lenzner et al., 2022).

All biogeographical studies depend on accurate assessment of not only richness patterns among known species but on the plant species themselves having been described. Arunachal Pradesh is particularly under‐studied with fewer native species recorded than the neighbouring Bhutan region, despite being larger (Rana, Price, et al., 2019). However, Bhutan and Jammu & Kashmir's floras are now relatively well known, and our own on‐site sampling of the flora of these specific regions gives concordant results with those based on the literature survey we report here (Rana et al., 2022; Rana, Gross, et al., 2019). Hence, we focused on these two regions. We find that warmer and wetter areas harbour relatively more alien species and suggest that filtering slows, or even may prevent, many alien species from range expansions. Our findings resemble those for geographical gradients north of Himalaya in China, where a latitudinal gradient in alien richness is tempered by especially high species numbers along the coasts, reflecting points of introduction (Li & Shen, 2020).

In summary, we attribute the high numbers of established alien plant species at low to mid elevations to a combination of climate at these elevations favouring high species diversity in general, and many introductions occurring at these elevations. Mid‐elevation peaks are found when we restrict our analysis to alien floras with temperate affinities, which we attribute to a similar combination of introductions of species from temperate regions, and climates favourable to temperate species. Elsewhere, exceptions to declines of alien species richness with elevation, such as a mid‐elevation peak observed in the Canary Islands (Alexander et al., 2011; Haider et al., 2010), have been linked to more favourable climates at these elevations (higher precipitation), as well as human disturbance, rather than site of introduction. In the case of the Himalaya, a role of introduction is likely, given many early settlements at 1500 m (e.g., at least 4500 years ago in the Kashmir valley, Yatoo et al., 2020). We now turn to consider factors affecting alien distributions in more detail.

### 4.2 Climatic control

Climatic affinities of species in their native range correlate with successful establishment (Hayes & Barry, 2008; Sax, 2001). In our case regional source pools (i.e., tropical/temperate affinities of genera) match both elevational and geographical distributions of alien species in the Himalaya and imply climatic controls on establishment. Elsewhere, a similarity of climatic niche between related alien species and native species has been inferred from an increase in phylogenetic clustering when alien species are added to native species, expected because closely related species are expected to share similar niches (Qian & Sandel, 2022). In the Himalaya, addition of alien species does not increase the clustering metric. In this case, increased clustering may not be expected, even if climatic affinity is an important correlate of establishment, because many species from the Americas and other distant locations are likely to share climatic niches by convergence, rather than shared ancestry (Qian et al., 2022).

Alien and native richness patterns are correlated with each other, and both are correlated strongly with climatic variables in the Himalaya (Tables S1 and S4). Sax (2001) noted that climatic correlations of alien species cannot result from speciation/extinction dynamics and instead may reflect direct climatic controls (e.g., productivity) on the number of species that can persist in an area, or possibly the number available to be introduced from climatically similar source areas.

### 4.3 Directional filtering

In addition to favourable climates, the location of introduction plus elapsed time since introduction have been invoked to explain distributions of alien species along mountainsides (Alexander et al., 2011; Haider et al., 2010; Marini et al., 2013). Across 10 elevational gradients in temperate regions and in tropical Hawaii, Alexander et al. (2011) demonstrated a widespread pattern of declines in alien species richness with elevation, associated with a nested pattern, whereby higher elevation alien species are subsets of low elevation alien species. They interpreted the declines to be a consequence of introductions occurring primarily at low elevations, with only some generalist species able to expand ranges to higher elevations (directional filtering).

Our findings from the Himalaya support this pattern. First, especially among those species with tropical affinities, we observe steeper declines in alien species than native species along the elevational gradient (Figure 2), which can be explained if introductions primarily occurred in lowlands, and it takes time to expand into novel climates. Second, we find peaks in alien species richness at about 1000 m below those for native richness (Figure 2), which applies separately for both groups with tropical and temperate affinities, suggesting that with time they could expand their ranges upslope. Alien species with temperate affinities show a peak at mid elevations, but as noted above this likely reflects introductions occurring at these elevations (e.g., *Prunus*, *Ranunculus*). Finally, we find a pattern of nestedness as one proceeds from base to higher elevations (Figure 6), matching the main result of Alexander et al. (2011).

### 4.4 Limits to range expansions

Directional filtering implies an inferred time delay in range expansions by aliens, which may reflect ability to enter different climates, especially in the face of competition from native species, and previously established alien species. Even for native species, the crossing of the freezing line appears to be difficult (Rana et al., 2022): relatively few genera contribute the bulk of the species above the freezing line, as indicated by the high phylogenetic clustering metric of native species at higher elevations (Figure 5), i.e., speciation rather than dispersal contributes importantly to native species richness at high elevations, an option yet to be realised by alien species.

Other features that could contribute to delayed range expansions are lower disturbance at higher elevations, or relatively high numbers of native species. Both these may operate at relatively small spatial grain sizes, but at the larger grain sizes of this study, we find patterns inconsistent with either of these factors, and instead propose climate and incumbency are the main limiting factors (see further discussion in the supplement). In the plains of India at a spatial scale of 700 m^2^, richness along a moisture gradient from dry scrub to moist evergreen forest is affected by high levels of disturbance and introductions, both of which are highest in native deciduous forests (Mungi et al., 2021). The spatial grain of that work and a focus on habitat rather than region differ from the present study.

The top three globally invasive plant species listed by Turbelin et al. (2017) are all found in the Himalaya and cover large areas. While it may be the case that on a regional scale, alien species increase species richness (Figure S7, Qian & Sandel, 2022; Stohlgren et al., 2003), at local grain sizes, this is not the case. Swathes of *Lantana camara*, *Ageratum conyzoides* and *Parthenium hysterophorus* cover many Himalayan locations, especially below 2000 m (Chaudhary et al., 2021; Lamsal et al., 2018; Mungi et al., 2020). Further expansion of *Lantana* and other alien species is to be expected (Lamsal et al., 2018; Mungi et al., 2018). Like the Himalayan region, alpine regions of America and Europe are also relatively free of alien species but likely to be increasingly invaded over time (Alexander et al., 2016). Our inference of a role for directional filtering implies range expansions are taking time to overcome, which we attribute to climate, and presumed climate‐associated competition. In consequence, we expect ongoing invasions of higher altitudes to be accelerated by climate change (Wani et al., 2023).

## AUTHOR CONTRIBUTIONS

**Suresh K. Rana:** Conceptualization (equal); data curation (lead); formal analysis (lead); funding acquisition (lead); investigation (lead); methodology (equal); supervision (supporting); visualization (equal); writing – original draft (equal). **Bhawana Dangwal:** Data curation (equal); investigation (equal); resources (equal). **Gopal S. Rawat:** Conceptualization (equal); methodology (equal); supervision (equal). **Trevor D. Price:** Conceptualization (equal); formal analysis (equal); methodology (supporting); supervision (supporting); visualization (supporting); writing – review and editing (equal).

## CONFLICT OF INTEREST STATEMENT

The authors declare no conflict of interest.

## Supporting information


Appendix S1.

Appendix S2.

Appendix S3.

## Data Availability

Data on alien species distributions are obtained from regional floras and the published literature in the Himalaya (Appendix S2) and is provided as a supplement with this paper (Appendix S3).
