# Supplementary material for: Constructing a database of alien plants in the Himalaya to test patterns structuring diversity
Source: Ecol Evol. 2024 Feb 9;14(2):e10884. doi: 10.1002/ece3.10884 (PMC10857928; doi:10.1002/ece3.10884)
Supplement: Supplementary file 1 — Appendix S1. [file ECE3-14-e10884-s002.docx]

## Supporting Information

**Constructing a database of alien plants in the Himalaya to test patterns structuring diversity**

**Suresh K. Rana^1^, Bhawana Dangwal^1^, Gopal S. Rawat^2^, Trevor D. Price^3^**

**Fig S1** Testing models of biotic interactions on Himalayan plants.

**Fig S2** Elevational patterns of disturbance in the Himalayan landscape.

**Fig S3** Elevational patterns of naturalized, cultivated and invasive alien species in the Bhutan region and Jammu & Kashmir.

**Fig S4** Elevational patterns of trees, shrubs and herbs for the native and alien species in Jammu & Kashmir and the Bhutan region.

**Fig S5** Elevational patterns of Nearest Taxon Index in Jammu & Kashmir and the Bhutan region in the Himalaya.

**Fig S6** Elevational patterns of species to genus ratios separated into species with tropical and temperate affinities.

**Fig S7** The proportion of aliens is high where native richness is high.

**Table S1** Correlation matrix for major variables along elevational gradients.

**Table S2** Nestedness metric (NODFc) for alien and native species along elevational gradients.

**Table S3** Multiple regression of alien species richness along elevational gradients in Jammu & Kashmir and the Bhutan region.

**Table S4.** Multiple regression of total alien species richness for three variables i.e. elevation, region and number of natives and their interactions.

**Methods S1** Assessing impact of native species richness on alien establishment.

**Fig. S1** (A) Himalayan data based on 1140 grid cells of 0.2^o^. Horizontal line is the average proportion of natives in the entire dataset. (B) Plot of 10 regression slopes, using the null model code from Beaury et al. (2020), based on the GLM approach (above) and LM approach (below), where mean proportion of natives, *P(n)* = 0.9 (horizontal line). In the GLM case, the median slope of 5,000 samples = -0.7 x 10^-3^ and 70% of the regression slopes were negative. In the LM case the median slope of the 5,000 samples = -0.008 x 10^-3^ and 52% of the regression slopes were negative. (C) Proportion of negative regression slopes in 500 samples, as a function of the lower bound on the total number of species, where the maximum is 165 (computed with lower bounds of 1, 10, 20 and 50 species). The GLM gives higher error rates than the LM, illustrated for two values of average proportion of natives at a site.


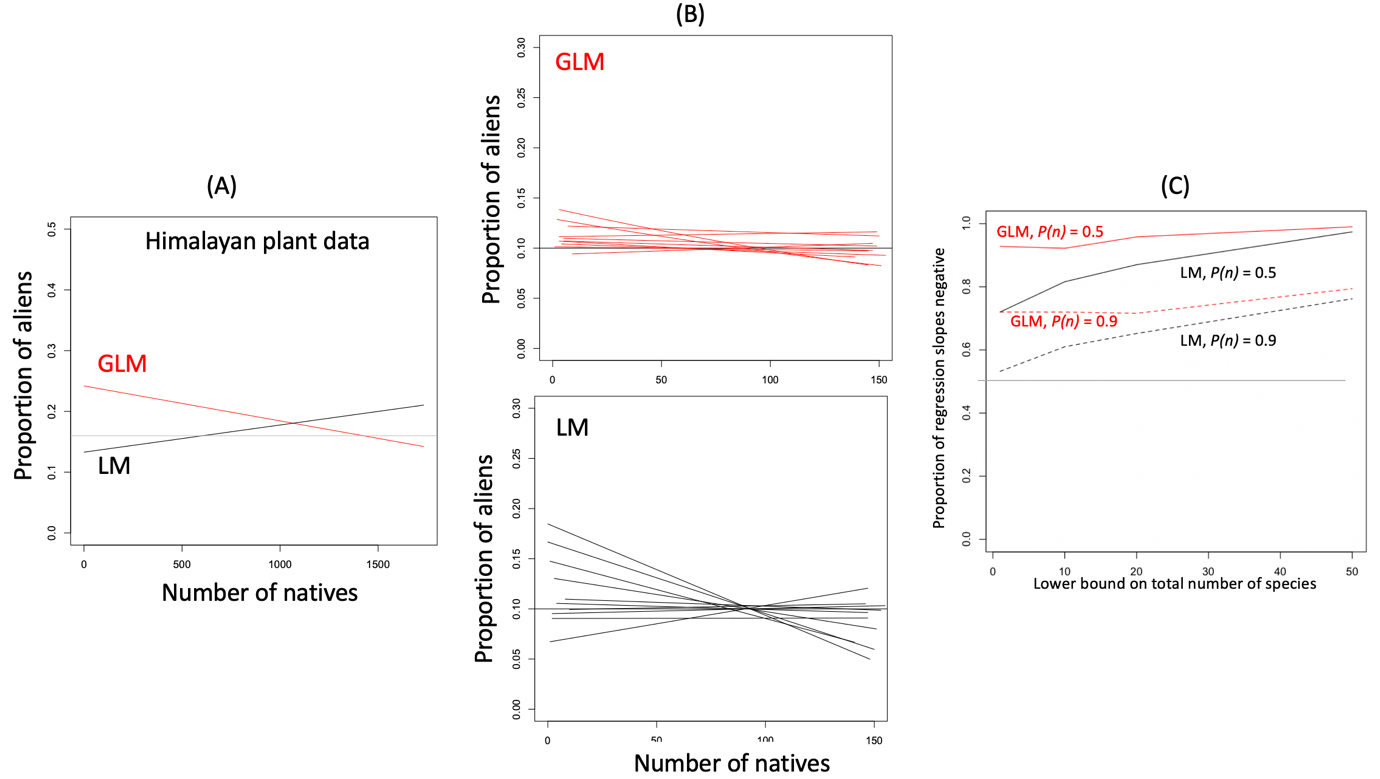


**Fig. S2** Elevational patterns of disturbance in the Himalayan landscape.

**Fig. S3** Elevational patterns of naturalized, cultivated, and invasive alien species in the Bhutan region and Jammu & Kashmir. Note that cultivated and naturalized form mutually exclusive datasets, whereby many cultivated species contain naturalized populations. Invasive species form a subset of naturalized species and are labelled as such based on the Global Invasive species database, rather than applying to the Himalaya.

**Fig. S4** Elevational patterns of trees, shrubs and herbs for the native and alien species in Bhutan region and Jammu & Kashmir.

**Fig. S5** Elevational patterns of Nearest Taxon Index in Jammu & Kashmir and Bhutan region in the Himalaya (blue natives, orange aliens, dashed total).

**Fig. S6** Elevational patterns of species to genus ratios separated into species with tropical and temperate affinities.

**Fig. S7** *Left:* The proportion of aliens is high where native richness is high. *Right:* The number of aliens increases with disturbance, which is higher in the west. Note that within region disturbance is correlated with elevation (Figure S2).

**Table S1** Correlation matrix for the data in Tables S3 and S4, N = 39 elevational bands.

**Table S2** Nestedness metric (NODFc) for aliens and natives, separated into those with tropical, temperate and all species combined along elevational gradients in east and northwest Himalaya, and for all low elevation species from west to east (Geography).

| **Group** | **Bhutan region** | | **Jammu & Kashmir** | | **Geography** | |
| --- | --- | --- | --- | --- | --- | --- |
|  | **Natives** | **Aliens** | **Natives** | **Aliens** | **Natives** | **Aliens** |
| All angiosperms | 0.57 | 1.06 | 0.64 | 0.96 | 0.74 | 0.89 |
| Tropical species | 0.95 | 1.29 | 0.96 | 1.18 | 0.83 | 0.97 |
| Temperate species | 0.63 | 0.95 | 0.75 | 0.98 | 0.76 | 1.1 |

**Table S3** Partial regression coefficients (β) from multiple regressions of total alien species richness in the 21 elevational belts of 200m below 4,000m from the Bhutan region (coded as 0) and 18 elevational belts from Jammu & Kashmir (coded as 1) combined (i.e., N = 39). Disturbance is average 2009 Human footprint in the belt (Venter et al. 2016), climate is the average first principal component score from the correlation matrix of the precipitation and temperature variables in Figure 4. Independent variables are all scaled to unit variance. Second and third columns drop elevation and number of native species respectively from the full model. For the correlation matrix, see Table S1.

| **Variable** | **β** | **s.e.** | **P value** | **β** | **s.e.** | **P value** | **β** | **s.e.** | **P value** |
| --- | --- | --- | --- | --- | --- | --- | --- | --- | --- |
| Disturbance | -1.7 | 5.5 | 0.76 | 7.7 | 6.3 | 0.23 | -10.5 | 5.0 | 0.23 |
| Climate | 32.3 | 14.4 | 0.03 | 80.1 | 11.2 | 0.00000 | 66.4 | 15.4 | 0.00010 |
| Elevation | -51.6 | 12.1 | 0.0002 | - | - | - | -52.7 | 15.1 | 0.00140 |
| Region | 52.9 | 12.0 | 0.0001 | 67.9 | 14.1 | 0.00003 | 67.9 | 14.1 | 0.00003 |
| No. natives | 72.6 | 16.0 | 0.0001 | 74.0 | 19.7 | 0.00064 | - | - | - |

**Table S4** Partial regression coefficients (β) from multiple regressions of total alien species richness for three variables i.e. elevation, region and number of natives and their interactions. Data as in Table S3. Second columns drop native species from the full model. For the correlation matrix, see Table S1.

| **Parameter** | **β** | **s.e.** | **P value** | **β** | **s.e.** | **P value** |
| --- | --- | --- | --- | --- | --- | --- |
| Elevation | -0.45 | 0.10 | 0.0001 | -0.90 | 0.04 | 0.0000 |
| Region | 0.10 | 0.26 | 0.0001 | -0.38 | 0.09 | 0.0001 |
| Natives | -0.74 | 0.15 | 0.0000 | - | - | - |
| Elevation:Region | 0.14 | 0.21 | 0.50 | 0.40 | 0.09 | 0.0001 |
| Elevation:Natives | -0.14 | 0.12 | 0.25 | - | - | - |
| Natives:Region | 0.06 | 0.31 | 0.85 | - | - | - |
| Elevation:Natives:Region | 0.55 | 0.24 | 0.03 | - | - | - |

**Methods S1** Assessing impact of native species richness on alien establishment

A meta-analysis finds native and alien species richness are positively correlated across all spatial scales, but particularly so at the spatial grain of this study (100s km^2^) (Peng *et al.* 2019). Despite this positive association, Beaury et al. (2020) proposed that one could test for biotic resistance (i.e., native species inhibiting establishment of aliens) by regressing the *proportion* of aliens in a local site against number of natives ($\frac{a}{n+a}$ *~ n* where *a* is the number of aliens in a site, and *n* is number of natives). Beaury et al. (2020) argued that a negative slope would support biotic resistance*.* To do this, Beaury et al. (2020) make a strong assumption, which is that the number of ‘niches’ in a location (on the scale of their study, 25 m^2^ – 5400 m^2^) is filled and given by the sum of natives and aliens. Unless the number of individuals is small and sets an upper limit on the number of species (Fridley *et al.* 2004) this is a model of interactions between species, whereby an alien species usurps a native, and a negative correlation implies natives usurp proportionately fewer natives in locations with many native species. If instead, alien species increase local species richness rather than displace native species (Stohlgren *et al.* 2008) negative correlations are expected in the absence of any species interactions. For example, if a single alien species is found everywhere, the correlation of proportion of alien species with number of native species would be -1.

The use of proportions has further been criticized for generating a spurious negative correlation, given the number of alien species is in both the numerator and the denominator (Pearson 1896, additional references in Beaury et al. 2020; Muthukrishnan 2021). Beaury et al. 2020 (p.4 in their supplement) presented helpful code for their null model, which we further analyze here (Figure S6). When estimated using the general linear model which they employed (glm(cbind(*a,n*) ~ *n,* family = binomial), the median slope is negative. This suggests caution in the interpretation of the ubiquitous negative slopes uncovered by Beaury et al. (2020, 2021) in the empirical data. A simple regression of the proportion of natives against natives: lm(*a/(a+n)~n)* results in a smaller bias. The essential difference between the two models is that in the GLM, each site is weighted by the number of species in the site, hence much greater weighting is given to sites with many species. Assuming each species is independent, a GLM may be appropriate when variation in sample size depends on sampling effort, but it is not clear that is the case when variation reflects true differences in numbers of natives, which is the thrust of the hypothesis.

In both cases the bias is larger when sites with small total numbers are excluded, and when the proportion of natives approaches 0.5 (Figure S6). As noted by Muthukrishnan (2021) for a fixed total, the numbers of aliens and natives must be perfectly negatively correlated, and it appears that especially high numbers of negative correlations in null models result whenever the (co)variance between sites is reduced so that covariances within each fixed total dominate.

Based on our investigations of the null model, we plotted proportion of natives against natives based on the linear model. The proportion shows a positive slope (Figures S1, S7), opposite to null model biases. Hence the data provide no support for the premise that locations with large numbers of native species are those that inhibit establishment of aliens. However, our analysis is at a scale of regions (g diversity), whereas species interactions should operate on small scales (a diversity). Hence, our interpretation that the positive correlation negates a role for biotic interactions depends on additional assumptions which are either (1) That turnover across elevational bands of 200m is the prime determinant of local conditions or (2) Turnover across sites (b diversity) is similar in different bands (because a = g/b, so with fixed b, a is proportional to g).

**References**

Beaury, E.M., Finn, J.T., Corbin, J.D., Barr, V. & Bradley, B.A. (2020). Biotic resistance to invasion is ubiquitous across ecosystems of the United States. *Ecology Letters*, 23, 476–482.

Beaury, E.M., Finn, J.T., Corbin, J.D. & Bradley, B.A. (2021). Habitat covariates do not artificially cause a negative correlation between native and non‐native species richness. *Ecology Letters*, 24, 1735–1737.

Fridley, J.D., Brown, R.L. & Bruno, J.F. (2004). Null models of exotic invasion and scale-dependent patterns of native and exotic species richness. *Ecology,* 85, 3215–3222.

Muthukrishnan, R. (2021). The relationship between native species richness and exotic species richness or occurrence will always be negative when the total number of species is accounted for in statistical models: A response to Beaury et al. *Ecology Letters*, 24, 1732–1734.

Pearson, K. (1897) Mathematical contributions to the theory of evolution—on a form of spurious correlation which may arise when indices are used in the measurement of organs. *Proceedings of the Royal Society of London* 60.359-367.

Peng, S., Kinlock, N.L., Gurevitch, J. & Peng, S. (2019). Correlation of native and exotic species richness: a global meta‐analysis finds no invasion paradox across scales. *Ecology,* 100, e02552.

Stohlgren, T.J., Barnett, D.T., Jarnevich, C.S., Flather, C. & Kartesz, J. (2008). The myth of plant species saturation. *Ecology Letters,* 11, 313–322.
