# Supplementary material for: Constructing a database of alien plants in the Himalaya to test patterns structuring diversity
Source: Ecol Evol. 2024 Feb 9;14(2):e10884. doi: 10.1002/ece3.10884 (PMC10857928; doi:10.1002/ece3.10884)
Supplement: Supplementary file 2 — Appendix S2. [file ECE3-14-e10884-s003.docx]

**Appendix-1. Data Sources**

1. **Research articles**

Ahmad, M., Uniyal, S., Singh, R., 2018. Patterns of alien plant species richness across gradients of altitude: Analyses from the Himalayan state of Himachal Pradesh. Tropical Ecology 59.

Araf, M., Kumar, S., Parihar, J., Hamal, I.A., n.d. Seed Biology of an Invasive Weed–*Euphorbia geniculata* Ortega from North- west Himalaya (India) 3.

Baral, S., Adhikari, A., Khanal, R., Malla, Y., Kunwar, R., Basnyat, B., Gauli, K., Acharya, R., 2017. Invasion of alien plant species and their impact on different ecosystems of Panchase Area, Nepal. Banko Janakari 27. <https://doi.org/10.3126/banko.v27i1.18547>

Barua, I., Deka, J., Devi, M., Deka, R., Moran, J., 2017. Weeds as Emerging Threat to Biodiversity:A Consequence of Spread of Ludwigia peruviana in Dhansiri and Kopili Catchment Areas of Assam, North East India. Current Science 112, 1904–1914. <https://doi.org/10.18520/cs/v112/i09/1904-1914>

Bhat, J., Kumar, M., Negi, A., Todaria, N., 2012. Acacia dealbata Link. (Silver Wattle), an invasive species growing in high altitudes of the Himalaya. undefined.

Bhatt, J., Singh, J.S., Singh, S., Tripathi, R., Kohli, R., 2012. Plant invasions in J & K In: Invasive Alien Plants An Ecological Appraisal for the Indian Subcontinent.

Bhatt, M., Panta, S., Singh, S., 2010a. Impact of Weed Management Practices on Biomass and Grain Yield of Two Varieties of Paddy in Lowland Area of Far-Western Nepal. Nepal Journal of Science and Technology 9. <https://doi.org/10.3126/njst.v9i0.3157>

Bhatt, M., Tewari, A., Singh, S., 2010b. Floristic Composition of Weeds in Paddy Fields in Mahendranagar, Nepal. Ecoprint: An International Journal of Ecology 16. <https://doi.org/10.3126/eco.v16i0.3468>

Bhattacharjya, D.K., Borah, P.C., 2008. Medicinal weeds of crop fields and role of women in rural health and hygiene in Nalbari district, Assam. IJTK Vol.7(3) [July 2008].

Bhattarai, K.R., Måren, I.E., Subedi, S.C., 2014. Biodiversity and invasibility: Distribution patterns of invasive plant species in the Himalayas, Nepal. Journal of Mountain Science 11, 688–696. <https://doi.org/10.1007/s11629-013-2821-3>

Bughani, I., Rajwar, G., 2005. Primary Productivity and the Impacts of the Exotic Weed Eupatorium Glandulosum in a Montane Grassland of Garhwal Himalaya. The Environmentalist 25, 31–38. <https://doi.org/10.1007/s10669-005-3094-3>

Chaudhary, V.P., Kumar, N., Srivastva, A., 2006. Crop-weed competition in onion (Allium cepa) under mid-hill condition of North-west Himalayas. Indian Journal of Agricultural Sciences 76, 744–746.

Dangwal, L., Singh, A., Singh, T., Sharma, A., 2011. Common weeds of kharif crops of block sunderbani district Rajouri (Jammu and Kashmir). undefined.

Dar, P.A., Reshi, Z.A., n.d. Do alien plant invasions cause biotic homogenization of terrestrial ecosystems in the Kashmir Valley, India? 13.

Das, A., 2002. Survey of naturalised exotics in the flora of Darjeelng Hills, West Bengal (India). Journal of Economic and Taxonomic Botany 26, 31–36.

Das, M., Purkayastha, J., Bauer, A., Sengupta, S., 2011. Hemidactylus flaviviridis Rüppell, 1835 (Sauria: Gekkonidae) an invasive gecko in Assam. North-Western Journal of Zoology 7, 98–104.

Datta, A., Kühn, I., Ahmad, M., Michalski, S., Auge, H., 2017. Processes affecting altitudinal distribution of invasive Ageratina adenophora in western Himalaya: The role of local adaptation and the importance of different life-cycle stages. PLoS ONE 12, e0187708. <https://doi.org/10.1371/journal.pone.0187708>

Debnath, A., Debnath, B., 2017. Diversity, Invasion Status and Usages of Alien Plant Species in Northeastern Hilly State of Tripura: A Confluence of Indo-Barman Hotspot. American Journal of Plant Sciences 8, 212–235. <https://doi.org/10.4236/ajps.2017.82017>

Debnath, A., Pauland, R., Debnath, B., 2018. Effects of the Invasive shrub, Chromolaena, odorata on Soil Properties in the Atharamura forest ecosystem: Indian Himalayan state of Tripura, North East India. Vegetos 31, 77–90. <https://doi.org/10.5958/2229-4473.2018.00059.9>

Debnath, B., Debnath, A., Paul, C., 2015. DIVERSITY OF INVASIVE PLANT SPECIES IN TRISHNA WILDLIFE SANCTUARY, TRIPURA, NORTHEAST INDIA BIMAL DEBNATH*, AMAL DEBNATH AND CHIRANJIT PAUL PLANT DIVERSITY AND FOREST BIOTECHNOLOGY LAB, DEPARTMENT OF FORESTRY AND BIODIVERSITY. Life Sciences Leaflets 70, 9–21.

Deka, J., Barua, I., 2015. Problem weeds and their management in the North-East Himalayas. undefined.

Dixit, J., 2008. Field Evaluation of Power Weeder for Rain-fed Crops in Kashmir Valley. Ama, Agricultural Mechanization in Asia, Africa & Latin America 39, 53–56.

Dogra, K., Kohli, R., Sood, S., 2009. An assessment and impact of three invasive species in the Shivalik hills of Himachal Pradesh, India. International Journal of Biodiversity and Conservation 1, 4–10.

Dogra, K., Sood, S., Sharma, R., 2011. Distribution, Biology and Ecology of Parthenium hysterophorus L. (Congress Grass) an invasive species in the North-Western Indian Himalaya (Himachal Pradesh) 5(11).

Everard, M., Gupta, N., Chapagain, P.S., Shrestha, B.B., Preston, G., Tiwari, P., 2018. Can control of invasive vegetation improve water and rural livelihood security in Nepal? Ecosystem Services 32, 125–133. <https://doi.org/10.1016/j.ecoser.2018.07.004>

Ganie, A., Reshi, Z., Wafai, B.A., 2011. A brief appraisal of genus Potamogeton L. in the Kashmir Valley. Invasive Alien Plants: An Ecological Appraisal for the Indian Subcontinent 126–130.

Gupta, V., Singh, M., Kumar, A., Sharma, B.C., Kher, D., 2014. Effect of different weed management practices in urdbean (Vigna Mungo L. Hepper) under sub-tropical rainfed conditions of Jammu, India. Legume Research - An International Journal 37, 424. <https://doi.org/10.5958/0976-0571.2014.00654.7>

Husain, D., 2016. Morphology and Anatomy of Dwarf Shoots of Some Exotic Species of Pinus Linn. in Kumaon Hills, Western Himalayas. International Journal of Current Microbiology and Applied Sciences 5, 215–223. <https://doi.org/10.20546/IJCMAS.2016.509.025>

Inderjit, Pergl J, van Kleunen M, Hejda M, Babu CR, Majumdar S, Singh P, Singh SP, Salamma S, Rao BRP, Pyšek P. 2018. Naturalized alien flora of the Indian states: biogeographic patterns, taxonomic structure and drivers of species richness. *Biological Invasions* **20**: 1625–1638.

Ingole, N.A., Nain, A.S., Kumar, P., Chalal, R., 2018. Monitoring and Mapping Invasive Aquatic Weed Salvinia molesta using Multispectral Remote Sensing Technique in Tumaria Wetland of Uttarakhand, India. Journal of the Indian Society of Remote Sensing 46, 863–871. <https://doi.org/10.1007/s12524-018-0764-4>

Jaryan, V., Datta, A., Uniyal, S., Kumar, A., Gupta, R.C., Singh, R., 2013a. Modelling potential distribution of Sapium sebiferum– an invasive tree species in western Himalaya. Current science 105, 1282–1288.

Jaryan, V., Uniyal, S., Kumar, A., Gupta, R.C., Parkash, O., Singh, R., 2013b. Distribution Characteristics of Sapium sebiferum (L.) Roxb. — An Invasive Tree Species in Himachal Pradesh, Western Himalaya. Proceedings of the Indian National Science Academy 79, 215–234.

Jaryan, V., Uniyal, S.K., Gupta, R.C., Singh, R.D., 2014. Litter Fall and Its Decomposition in *Sapium sebiferum* Roxb.: An Invasive Tree Species in Western Himalaya. International Journal of Ecology 2014, e142429. <https://doi.org/10.1155/2014/142429>

Jaryan, V., Uniyal, S.K., Gupta, R.C., Singh, R.D., 2013c. Alien flora of Indian Himalayan state of Himachal Pradesh. Environmental Monitoring and Assessment 185, 6129–6153. <https://doi.org/10.1007/s10661-012-3013-2>

Karmakar, N.C., Hazra, A., 2016. First evidences for induced pseudo-viviparous germination in Ageratina adenophora (Crofton weed), a common alien weed of Darjeeling Himalaya, India. Plant Science Today 3, 249–257. <https://doi.org/10.14719/pst.2016.3.3.234>

Karov, S., Ram, V., Rangappa, K., Singh, N.J., Aradhya, P., Ray, L.I.P., 2018. Weed Pressure on Growth and Yield of Groundnut (Arachis hypogaea L.) in Meghalaya, India. International Journal of Current Microbiology and Applied Sciences 7. <https://doi.org/10.20546/ijcmas.2018.703.328>

Khadka, A., 2017. Assessment of the perceived effects and management challenges of Mikania micrantha invasion in Chitwan National Park buffer zone community forest, Nepal. Heliyon 3, e00289. <https://doi.org/10.1016/j.heliyon.2017.e00289>

Khan, A., Arya, D., 2019. Impact of Climate Change on the Proliferation of Invasive Alien Plant Species in Almora district of Uttarakhand Himalaya.

Khuroo, A., 2012. Plant invasions in J & K, India.

Khuroo, A., Malik, A., Reshi, Z., Dar, G.H., 2010a. From ornamental to detrimental: Plant invasion of Leucanthemum vulgare Lam. (Ox-eye Daisy) in Kashmir valley, India. Current Science 98, 600–602.

Khuroo, A., Weber, E., Malik, A., Dar, G.H., Reshi, Z., 2010b. Taxonomic and biogeographic patterns in the native and alien woody flora of Kashmir Himalaya, India. Nordic Journal of Botany 28, 685–696. <https://doi.org/10.1111/j.1756-1051.2010.00750.x>

Khuroo, A.A., Rashid, I., Reshi, Z., Dar, G.H., Wafai, B.A., 2007. The alien flora of Kashmir Himalaya. Biological Invasions 9, 269–292. <https://doi.org/10.1007/s10530-006-9032-6>

Khuroo, A.A., Weber, E., Malik, A.H., Reshi, Z.A., Dar, G.H., 2011. Altitudinal distribution patterns of the native and alien woody flora in Kashmir Himalaya, India. Environmental Research 111, 967–977. <https://doi.org/10.1016/j.envres.2011.05.006>

Kishore, K., Monika, N., Rinchen, D., Pandey, B., Singh, R., Rahman, H., Kumar, A., 2012. Evaluation of Exotic Mandarin Germplasm under Mid-Hills of Sikkim Himalayan Region. Indian Journal of Plant Genetic Resources 25, 153–156.

Kohli, R., Dogra, K., Batish, D., Singh, H.P., 2009. Impact of Invasive Plants on the Structure and Composition of Natural Vegetation of Northwestern Indian Himalayas1. Weed Technology 18, 1296–1300. [https://doi.org/10.1614/0890-037X(2004)018[1296:IOIPOT]2.0.CO;2](https://doi.org/10.1614/0890-037X(2004)018%5b1296:IOIPOT%5d2.0.CO;2)

Kosaka, Y., Saikia, B., Mingki, T., Tag, H., Riba, T., Ando, K., 2010. Roadside Distribution Patterns of Invasive Alien Plants Along an Altitudinal Gradient in Arunachal Himalaya, India. Mountain Research and Development 30, 252–258. <https://doi.org/10.1659/MRD-JOURNAL-D-10-00036.1>

Kumar, B., Srivastava, S., Rawat, A., 2015. Intra-Specific Variation of Precocene I in the Wild Population of Ageratum conyzoides L. from the Western Himalayas. JPC - Journal of Planar Chromatography - Modern TLC 28, 391–394. <https://doi.org/10.1556/1006.2015.28.5.8>

Kumar, P., Pant, M., Gcs, N., 2011. Lantana mulching for soil fertility improvement, soil and water conservation and crop yield enhancement in rainfed rice in the Kumaun Hills. Invasive Alien Plants: An Ecological Appraisal for the Indian Subcontinent 282–291.

Maharjan, S., Joshi, S., Shrestha, B., Devkota, A., Jha, P., 2014. Life History Traits and Invasion Success of Parthenium hysterophorus L. in Kathmandu Valley, Nepal. Journal of Science and Technology 15, 31–38. <https://doi.org/10.3126/njst.v15i1.12007>

Mandal, G., Joshi, S.P., 2015. Estimation of above-ground biomass and carbon stock of an invasive woody shrub in the subtropical deciduous forests of Doon Valley, western Himalaya, India. Journal of Forestry Research 26, 291–305. <https://doi.org/10.1007/s11676-015-0038-8>

Mandal, G., Laboratory, E.R., of Botany, D., College, D. (pg), Dehradun, Uttarakhand, India, Joshi, S.P., Laboratory, E.R., of Botany, D., College, D. (pg), Dehradun, Uttarakhand, India, 2014. Phytogeography and Invasion Spread of Chromolaena odorata (L.) R.M. King et H. Rob. in the Western Himalaya, India. Botanica Pacifica 3, 35–46.

Masoodi, A., Khan, F., 2012. Invasion of alligator weed (Alternanthera philoxeroides) in Wular Lake, Kashmir, India. Aquatic Invasions 7. <https://doi.org/10.3391/ai.2012.7.1.016>

Mehraj, G., Khuroo, A.A., Qureshi, S., Muzafar, I., Friedman, C.R., Rashid, I., 2018. Patterns of alien plant diversity in the urban landscapes of global biodiversity hotspots: a case study from the Himalayas. Biodiversity and Conservation 27, 1055–1072. <https://doi.org/10.1007/s10531-017-1478-6>

Mungi, N., Coops, N., Ramesh, K., Rawat, G., 2018. How global climate change and regional disturbance can expand the invasion risk? Case study of Lantana camara invasion in the Himalaya. Biological Invasions 20, 1–15. <https://doi.org/10.1007/s10530-018-1666-7>

Negi, P.S., Hajra, P.K., 2007. Alien flora of Doon Valley, Northwest Himalaya. Current Science 92, 968–978.

Paudel, R., 2011. Insight into Invasive Species (Mikania micrantha), Its Control Measures and Programmes in Nepal. The Initiation 4, 115–119. <https://doi.org/10.3126/init.v4i0.5544>

Pradhanang, P.M., Elphinstone, J.G., Fox, R.T.V., 2000. Identification of crop and weed hosts of Ralstonia solanacearum biovar 2 in the hills of Nepal. Plant Pathology 49, 403–413. <https://doi.org/10.1046/j.1365-3059.2000.00480.x>

Prakash, V., Srivastva, A.K., 2006. Crop-Weed Competition Studies in Tomato (Lycopersicon esculentum) under Mid-Hills of North-West Himalayas. undefined.

Prakash, V., Srivastva, A.K., n.d. Crop-Weed Competition Studies in Gardenpea (Pisum sativum) under Mid-Hill Conditions of North-West Himalayas 5.

Princejayasimha, P., Baiswar, P., Kumar, R., Majumder, D., Patra, S., 2018. Pathogenicity of Rhizoctonia solani AG 1-IB on common weeds in Meghalaya. Indian Journal of Weed Science 50, 72. <https://doi.org/10.5958/0974-8164.2018.00016.3>

Princejayasimha, P., Baiswar, P., Kumar, R., Majumder, D., Patra, S., 2017. Pathogenicity of Sclerotium spp. on Common Weeds in Meghalaya, India. Indian Phytopathology 70. <https://doi.org/10.24838/ip.2017.v70.i4.76996>

Qurashi, M.A., Hassan, S.M., Shah, G.A., 2017. Effect of butachlor on Haematological profile of exotic carp, Cyprinus carpio var communis (Linn) of Manasbal lake of Kashmir valley. undefined.

Rai, R.K., Scarborough, H., Subedi, N., Lamichhane, B., 2012. Invasive plants – Do they devastate or diversify rural livelihoods? Rural farmers’ perception of three invasive plants in Nepal. Journal for Nature Conservation 20, 170–176. <https://doi.org/10.1016/j.jnc.2012.01.003>

Rana, M.C., Sharma, G.D., Sharma, A., Rana, S., 2004. Effect of weed management and fertility levels on rajmash (Phaseolus vulgaris) and associated weeds under dry temperate high hills in Himachal Pradesh. Indian Journal of Weed Science 36, 227–230.

Rana, S., 2002. Integrated weed management in pea (Pisum sativum L.) under Sangla valley conditions of Himachal Pradesh. Indian Journal of Weed Science 34, 204–207.

Rao, R.R., Sagar, K., 2011. Invasive alien weeds in the tropics: The changing pattern in the herbaceous flora of Meghalaya in north-east India. Invasive Alien Plants: An Ecological Appraisal for the Indian Subcontinent 189–198.

Rasheed, S., Khuroo, A.A., Hamid, M., Ganie, A.H., Malik, A.H., Dar, G.H., 2016. Phalaris canariensis L. (Poaceae): A new alien plant record for Kashmir Himalaya, India. Journal of Asia-Pacific Biodiversity 9, 94–96. <https://doi.org/10.1016/j.japb.2015.11.001>

Reshi, Z., Rashid, I., Khuroo, A., WAFAI, B., 2008. Effect of invasion by Centaurea iberica on community assembly of a mountain grassland of Kashmir Himalaya, India. Tropical Ecology 49.

Reshi, Z., Shah, M., Rashid, I., Rasool, N., 2011. Anthemis cotula L.: A highly invasive species in the Kashmir Himalaya, India. Invasive Alien Plants: An Ecological Appraisal for the Indian Subcontinent 108–125.

Reshi, Z., WAFAI, B., 2006. Demographic plasticity in relation to growth and resource allocation pattern in Anthemis cotula - An alien invasive species in Kashmir Himalaya, India. Applied Ecology and Environmental Research 4.

Roy, A., Bhattacharya, S., Ramprakash, M., Senthil Kumar, A., 2016. Modelling critical patches of connectivity for invasive Maling bamboo (Yushania maling) in Darjeeling Himalayas using graph theoretic approach. Ecological Modelling 329, 77–85. <https://doi.org/10.1016/j.ecolmodel.2016.02.016>

Sekar, K.C., Manikandan, R., Srivastava, S., 2012. Invasive Alien Plants of Uttarakhand Himalaya. Proceedings of the National Academy of Sciences, India Section B: Biological Sciences 82. <https://doi.org/10.1007/s40011-012-0040-2>

Sekar, K.C., Pandey, A., Giri, L., 2015. INVASIVE ALIEN PLANTS OF HIMACHAL PRADESH, INDIA. Indian Forester 141, 520–527.

Shah, M., Reshi, Z., 2014. Characterization of alien aquatic flora of Kashmir Himalaya: Implications for invasion management. Tropical Ecology 55, 143–157.

Shah, M., Reshi, Z., Lavoie, C., 2011. Predicting plant invasiveness from native range size: Clues from the Kashmir Himalaya. Journal of Plant Ecology 5, 167–173. <https://doi.org/10.1093/jpe/rtr021>

Shah, M., Reshi, Z., Rashid, I., 2008. Mycorrhizal source and neighbour identity differently influence Anthemis cotula L. invasion in the Kashmir Himalaya, India. Applied Soil Ecology 40, 330–337. <https://doi.org/10.1016/j.apsoil.2008.06.002>

Shah, M.A., Ali, M.A., Al-Hemaid, F.M., Reshi, Z.A., 2014. Delimiting invasive Myriophyllum aquaticum in Kashmir Himalaya using a molecular phylogenetic approach. Genetics and molecular research: GMR 13, 7564–7570. <https://doi.org/10.4238/2014.September.12.23>

Shah, M.A., Reshi, Z., Rashid, I., 2008. Mycorrhizosphere mediated Mayweed Chamomile invasion in the Kashmir Himalaya, India. Plant and Soil 312, 219–225. <https://doi.org/10.1007/s11104-008-9706-1>

Shah, M.A., Reshi, Z.A., Khasa, D., 2009. Arbuscular mycorrhizal status of some Kashmir Himalayan alien invasive plants. Mycorrhiza 20, 67–72. <https://doi.org/10.1007/s00572-009-0258-x>

Shankar, U., Yadav, A.S., Rai, J., Tripathi, R., 2011. Status of alien plant invasions in north-eastern region of India, in: Invasive Alien Plants: An Ecological Appraisal for the Indian Subcontinent. pp. 174–188. <https://doi.org/10.1079/9781845939076.0174>

Shil, S., Nath, D., 2015. Effect of Pendimethalin on Weed flora and Yield component of Brinjal under Agro-climatic condition of Tripura. undefined.

Shrestha, B., Dangol, D., 2015. Impact of Mikania micrantha H.B.K. Invasion on Diversity and Abundance of Plant Species of Chitwan National Park, Nepal. Journal of Institute of Science and Technology 19, 30. <https://doi.org/10.3126/jist.v19i2.13849>

Shrestha, K., Wilson, E., Gay, H., 2008. Ecological and Environmental Study of Eupatorium adenophorum Sprengel (Banmara) with Reference to its Gall Formation in Gorkha-Langtang Route, Nepal. Journal of Natural History Museum 20. <https://doi.org/10.3126/jnhm.v23i0.1848>

Shrestha, U.B., Sharma, K.P., Devkota, A., Siwakoti, M., Shrestha, B.B., 2018. Potential impact of climate change on the distribution of six invasive alien plants in Nepal. Ecological Indicators 95, 99–107. <https://doi.org/10.1016/j.ecolind.2018.07.009>

Singh, A., Sharma, A., 2014. Documentation of Invasive Alien Plants Species of Rupandehi District, Western Nepal. International Journal of Applied Sciences and Biotechnology 2. <https://doi.org/10.3126/ijasbt.v2i2.10370>

Srivastava, V., Griess, V.C., Padalia, H., 2018. Mapping invasion potential using ensemble modelling. A case study on Yushania maling in the Darjeeling Himalayas. Ecological Modelling 385, 35–44. <https://doi.org/10.1016/j.ecolmodel.2018.07.001>

Talapatra, K., Roy Das, A., Saha, A.K., Das, P., 2018. Culturable Root-Fungal Endophyte in Invasive Plants of Tripura, Northeast India: Seasonal Colonization and its Antimicrobial Activity. National Academy science letters.

Tantry, M.A., Shabir, S., Khan, R., Habib, A., Akbar, S., 2012. Determination of essential oil composition of Rosmarinus officinalis growing as exotic species in Kashmir Valley. Chemistry of Natural Compounds 47, 1012–1015.

Thapa, S., Chitale, V., Rijal, S.J., Bisht, N., Shrestha, B.B., 2018. Understanding the dynamics in distribution of invasive alien plant species under predicted climate change in Western Himalaya. PLOS ONE 13, e0195752. <https://doi.org/10.1371/journal.pone.0195752>

Timsina, B., Shrestha, B.B., Rokaya, M.B., Münzbergová, Z., 2011. Impact of Parthenium hysterophorus L. invasion on plant species composition and soil properties of grassland communities in Nepal. Flora - Morphology, Distribution, Functional Ecology of Plants 206, 233–240. <https://doi.org/10.1016/j.flora.2010.09.004>

Tshewang, S., Dendup, C., Tshering, P., Kristiansen, P., 2017. Weed research issues, challenges, and opportunities in Bhutan.

Verma, P., Kumar, V., Kaushik, P., Yadav, A., 2014. Bryophyte Invasion on Famous Archaeological Site of Ahom Dynasty ‘Talatal Ghar’ of Sibsagar, Assam (India). Proceedings of the National Academy of Sciences, India Section B: Biological Sciences 84. <https://doi.org/10.1007/s40011-013-0198-2>

Wani, G.A., Shah, M.A., Reshi, Z.A., Dar, M.A., 2018. Polyploidy determines the stage of invasion: clues from Kashmir Himalayan aquatic flora. Acta Physiologiae Plantarum 40, 58. <https://doi.org/10.1007/s11738-018-2629-4>

1. **Floras**

Babu, C. R. (1977). *Herbaceous flora of Dehradun*. CSIR Publications, New Delhi.

Bhellum, B. L., & Magotra, R. (2012). *A catalogue of flowering plants of Doda, Kishtwar and Ramban districts Kashmir Himalaya*. Bishan Singh Mahenddra Pal Singh Dehradun.

Blatter, E. (1927). *Beautiful flowers of Kashmir* (Vol.1-2). Staples and Staples Limited, Great Britain.

Chauhan, A. S., Singh, K. P., & Singh, D.K. (1996). *A contribution to the flora of Namdapha Arunachal Pradesh*. Botanical Survey of India, Calcutta.

Chowdhery, H. J., & Wadhwa B. M. (1984). *Flora of Himachal Pradesh* (Vol.1-3). Botanical Survey of India, Culcatta.

Chowdhery, H. J., Giri, G. S., & Pramanik, A. (2009). *Materials for the flora of Arunachal Pradesh* (Vol-3. Hydrocharitaceae-Poaceae). Botanical Survey of India, Culcatta.

Collet, H. (1921). *Flora Simlensis*. Thacker, Spink & Co., London.

Dhaliwal, D. S., & Sharma, M. (1999). *Flora of Kullu district (Himachal Pradesh)*. Bishen Singh Mahendra Pal Singh, Dehradun, India.

Dhar, U., & Kachroo, P. (1983). *Alpine flora of Kashmir Himalaya*. Scientific Publishers, Jodhpur, India.

Duthie, J. F. (1903-23). *Flora of the upper Gangetic plain and of The adjacent Siwalik and sub-Himalayan tracts*. Botanical Survey of India, Dehradun

Gaur, R. D. (1999). *Flora of the district Garhwal northwest Himalaya*. Transmedia, Srinagar U.P. India.

Ghosh, D. K., & Mallick, J. K. (2014). *Flora of Darjeeling Himalayas and foothills (Angiosperms)*. Bishen Singh Mahendrapal Singh, Dehradun.

Giri, G. S., Pramanik, A., & Chowdhery, H. J. (2008). *Materials for the flora of Arunachal Pradesh* (Vol-2. Astraceae-Ceratophyllaceae). Botanical Survey of India, Culcatta.

Grierson, A. J. C., & Long, D. G. (1983-87). *Flora of Bhutan (Including a record of plants from Sikkim)* (Vol. 1). Royal Botanical Garden, Edinburgh.

Grierson, A. J. C., & Long, D. G. (1991-2001). *Flora of Bhutan (Including a records of plants from Sikkim and Darjeeling)* (Vol. 2). Royal Botanic Garden, Edinburgh.

Gupta, R. K. (1968). *Flora Nainitalensis: A hand book of the flowering plants of Nainital*. Navayug Traders, New Delhi.

Hajra, P. K., & Badlodi, B. (1995). *Plant wealth of Nanda Devi Biosphere Reserve*. Botanical Survey of India, Calcutta.

Hajra, P. K., Verma, D. M., & Giri, G.S. (1996). *Materials for the Flora of Arunachal Pradesh* (Vol. 1). Botanical Survey of India, Culcatta.

Hara, H., & Williams, L. H. J. (1982). *An enumeration of the flowering plants of Nepal* (Vol. 1-3). Trustees of the British Museum, London

Kanjilal, U. N. (1928). *Forest flora of the Chakrata, Dehradun and Saharanpur forest division.* Government of India Press, Calcutta.

Kapur, S. K., & Sarin, Y. K. (1990). *Flora of Trikuta hills (Shri Vaishno Devi Shrine)*. Bishen Singh Mahendra Pal Singh, Dehradun, India.

Lal, K., & Rawat, G. S. *Flowering plants of Sirmour district: A catalogue*. (In Press).

Naithani, (1984). *Flora of Chamoli* (Vol. 1-2). Botanical Survey of India, Hawrah.

Noltie, H. J. (1994-2000). *Flora of Bhutan (Including a record of plants from Sikkim and Darjeeling)* (Vol. 3, Part 1-2). Royal Botanic Garden, Edinburgh.

Osmaston, A. E. (1994). *A Forest Flora of Kumaon*. Bishen Singh Mahinder Pal Singh, Dehradun.

Pearce, N. R., & Cribb, P. J. (2002). *Flora of Bhutan: Including a record of plants from Sikkim and Darjeeling* (Vol. 3, Part-3), *The Orchids of Bhutan*. Royal Botanic Garden, Edinburgh.

Sharma, B. M., & Jamwal, P. S. (1988). *Flora of upper Lidder valley of Kashmir Himalaya* (Vol. 1-2). Scientific Publishers, Jodhpur.

Sharma, B. M., & Kachroo, P. (1981). *Flora of Jammu and plants of neighbourhood*. Bishen Singh Mahendra Pal Singh, Dehradun.

Singh, H., & Sharma, M. (2006). *Flora of Chamba district Himachal Pradesh*. Bishan Singh, Mohinderpal, Dehradun

Singh, G., & Kachroo, P. (1994). *Forest flora of Pir Panjal range (Northwest Himalaya)*. Bishan Singh, Mohinder Pal Singh, Dehradun.

Singh, G., & Kachroo, P. K. (1987). *Forest flora of Srinagar*. Periodical Expert Book Agency, New Delhi.

Singh, S. K., & Rawat, G. S. (2000). *Flora of Great Himalayan National Park Himachal Pradesh*. Bishen Singh Mahinder Pal Singh Dehradun.

Strachey, R. (1974). *Catalogue of Kumaon Plants*. Periodical Experts Delhi

Swami, A., & Gupta, B.K. (1998). *Flora of Udhampur*. Bishen Singh Mahendra Pal Singh, Dehradun.

Uniyal, B. P., Sharma, J. R., Chaudhery U., & Singh. D. K. (2007). *Flowering plants of Uttarakhand.* Bishen Singh Mahendra Pal Singh, Dehradun.

Vij, S. P., Verma, J., & Kumar, C. S. (2013). *Orchids of Himachal Pradesh*. Bishen Singh Mahendrapal Singh, Dehradun.
